# Heidelberg-mCT-Analyzer: a novel method for standardized microcomputed-tomography-guided evaluation of scaffold properties in bone and tissue research

**DOI:** 10.1098/rsos.150496

**Published:** 2015-11-11

**Authors:** Fabian Westhauser, Christian Weis, Melanie Hoellig, Tyler Swing, Gerhard Schmidmaier, Marc-André Weber, Wolfram Stiller, Hans-Ulrich Kauczor, Arash Moghaddam

**Affiliations:** 1 Trauma and Reconstructive Surgery, Center of Orthopedics, Traumatology, and Spinal Cord Injury, Heidelberg University Hospital, Schlierbacher Landstraße 200a, Heidelberg 69118, Germany; 2 HTRG - Heidelberg Trauma Research Group, Schlierbacher Landstraße 200a, Heidelberg 69118, Germany; 3 Clinic of Diagnostic and Interventional Radiology (DIR), Heidelberg University Hospital, Im Neuenheimer Feld 110, Heidelberg 69120, Germany

**Keywords:** bone scaffold, bone tissue engineering, micro-computed tomography, scaffold characterization, bmp-7 (bone morphogenetic protein-7), human mesenchymal stem cells

## Abstract

Bone tissue engineering and bone scaffold development represent two challenging fields in orthopaedic research. Micro-computed tomography (mCT) allows non-invasive measurement of these scaffolds’ properties *in vivo*. However, the lack of standardized mCT analysis protocols and, therefore, the protocols’ user-dependency make interpretation of the reported results difficult. To overcome these issues in scaffold research, we introduce the Heidelberg-mCT-Analyzer. For evaluation of our technique, we built 10 bone-inducing scaffolds, which underwent mCT acquisition before ectopic implantation (T0) in mice, and at explantation eight weeks thereafter (T1). The scaffolds’ three-dimensional reconstructions were automatically segmented using fuzzy clustering with fully automatic level-setting. The scaffold itself and its pores were then evaluated for T0 and T1. Analysing the scaffolds’ characteristic parameter set with our quantification method showed bone formation over time. We were able to demonstrate that our algorithm obtained the same results for basic scaffold parameters (e.g. scaffold volume, pore number and pore volume) as other established analysis methods. Furthermore, our algorithm was able to analyse more complex parameters, such as pore size range, tissue mineral density and scaffold surface. Our imaging and post-processing strategy enables standardized and user-independent analysis of scaffold properties, and therefore is able to improve the quantitative evaluations of scaffold-associated bone tissue-engineering projects.

## 1. Background

Clinical routine in orthopaedic and trauma surgery demands bone substitutes for the treatment of critical size defects, prosthesis loosening, non-union, infection of bone or defects following tumour resection [1–3]. Research on scaffolds and substitutes for improving bone healing is increasing steadily. Especially, porosity, pore volume and surface structure seem to be crucial parameters for characterizing the properties of scaffolds that increase bone healing and regeneration [4,5]. Particularly, micro-computed tomography (mCT) analysis is capable of depicting common scaffold parameters (table 1), so mCT seems to be a reliable tool for the non-invasive analysis of scaffold structure.

**Table 1.** Outcome variables for scaffolds’ microstructure and architecture. The column ‘Program’ shows with which application the desired parameter can be evaluated. The following commercially available programs were evaluated: AM= Amira (FEI Visualization Sciences Group, Bordeaux, France) and CTA=CTAn (Skyscan). Our algorithm is represented by the acronym CA (=custom algorithm), arb. units, arbitrary units. Adapted from Bouxsein *et al.* [6].

abbreviation	variable	description	unit	program
TV	total volume	volume of the whole region of interest	metric	AM, CTA, CA
SV	scaffold volume	volume of the mineralized part of scaffold	metric	AM, CTA, CA
SS	scaffold surface	surface of the mineralized scaffold	metric	CA
SR=SS/SV	surface area to volume ratio	ratio of scaffold surface and scaffold volume	1/metric	CA
P=(TV−SV)/TV	porosity	ratio of the volume of voids and total volume	1/metric	CTA, CA
PN	pore number	total number of pores	(arb. units)	AM, CTA, CA
PSR	pore size range	shows number of pores in a set range of pore diameters	metric	CA
PV	pore volume	total volume of pores (PSR)	metric	AM, CTA, CA
PS	pore size	mean diameter of pores (PSR)	metric	CA
TMD	tissue mineral density	density of tissue mineral	HU	CA

In the last few years, mCT has become one of the most important methods for the evaluation of bone and its microstructure [7]. Recent studies show that data from mCT are significantly correlated with results from histomorphometry, the current gold standard for analysis of bone and tissue structure [8]. mCT could be used as an additional tool to histomorphometry in experimental and clinical settings—not only for the analysis of physiological bone tissue but also for the evaluation of bone scaffolds and substitutes [6,8]. The settings to achieve optimum performance of the mCT-device are well described and simplify the use of mCT in experimental designs [6].

Whereas the parameters and methods for analysis of physiological human or animal bone are well described, the evaluation of scaffolds is not completely standardized yet [6].

Thimm *et al.* [8] show that mCT-guided evaluation of ceramic scaffolds’ properties leads to the same results as their histomorphometrical analysis. Nevertheless, manual evaluation of porosity is time-consuming, and porosity is only one of many interesting structural aspects of bone scaffolds.

The aspects of the publications of Bouxsein *et al.* and Karageorgiou *et al.* combined with the idea of an ‘ideal’ bonegraft substitute-composition leads to a list of parameters for three-dimensional outcomes of bone substitute microstructure that should be evaluated and quantified to assess scaffold structure [4–6]. Accordingly, we adapted the outcome variables for scaffold microstructure and architecture as follows (table 1).

There are several commercially available programs for the analysis of mCT-data offering different bone structure parameters for evaluation (table 1). The investigation of optimum scaffold structure that includes the above-mentioned parameters differs from the evaluation of vital bone [6]. There are two major problems in mCT evaluation, especially in scaffold-associated projects: firstly, it is difficult to quantify the mentioned scaffold properties in a standardized way; and secondly, evaluation has to be fast, easy, efficient and specialized with regard to the scaffold’s microstructure [9,10]. Furthermore, several programs demand user action which makes the comparison and interpretation of generated data difficult [8–10].

Therefore, we developed the Heidelberg-mCT-Analyzer, an open-source software tool that allows specialized, fully automatic analysis of bone scaffold parameters as described in table 1. In the following, we present the software’s capabilities, the results of the data analysis using this algorithm and its user independence compared to other software.

## 2. Material and methods

In our standard tissue-engineering protocols, we perform *in vivo* bone formation of human mesenchymal stem cells (hMSCs) derived from iliac crest aspirate based on ectopic implantation of hMSC-coated scaffolds under stimulation with bone morphogenetic protein-7 (BMP-7) in severe combined immunodeficiency (SCID) mice (Charles River, Wilmington, MA, USA).

### 2.1 Patient demography

Bone marrow aspirate from iliac crest of five different donors (three males, two females) was taken for this study. Average age was 45.2±20.11 years. We did not match donors based on factors that could influence the quality of the aspirated bone marrow to provide the biological situation known from daily clinical routine within the experimental setting [11,12]. For the methods regarding hMSC characterization, isolation and cultivation, see the electronic supplementary material.

### 2.2 Scaffolds and scaffold coating

The scaffolds were made from 10 mg of sterilized phase-pure (more than 95%) granulated beta-tricalciumphosphate (bTCP; Ca_3_(PO_4_)_2_) with a granule size of 0.5–0.7 mm, a porosity of 60% and a pore size range of 100–500 μm with a number of undefined smaller pores of less than 100 μm in diameter (RMS Foundation, Bettlach, Switzerland) and 1×10^6^ hMSCs. The bTCP was stored within an Eppendorf vial (Eppendorf AG, Hamburg, Germany) and was dynamically coated with hMSCs in solution under the use of centrifugation (1000 r.p.m., 10 min, at room temperature). BMP-7 was added with a target concentration of 1 μg ml^−1^ and afterwards glued with fibrin–thrombin tissue glue (10 μl 1:15 PBS-diluted fibrin; 10 μl 1:50 PBS-diluted thrombin; TISSUCOL Duo S Immuno, Baxter U.S., Deerfield, IL, USA) to form a solid construct. We designed two identical constructs per donor—out of five donors, 10 constructs were built.

### 2.3 Implantation and explantation

**Figure 1.**
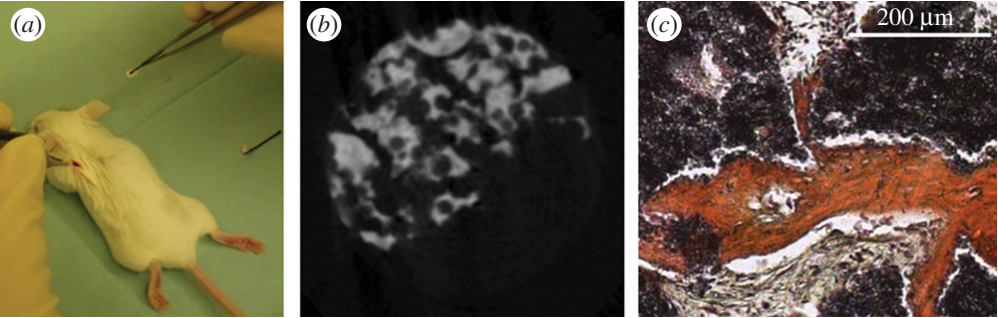
(*a*) Implantation of the scaffold at the back of the left upper extremity of a SCID mouse. (*b*) Representative slice of a three-dimensional reconstruction of a *μ*CT acquisition. Porous bTCP granules (white) were bonded together using fibrin–thrombin tissue glue (grey). (*c*) One of the constructs in HES-staining as an example. Red tissue: de novo bone formation. Black: bTCP. Grey: fibrous tissue.

The constructs were implanted ectopically at the back above the upper and lower extremities of three two- to six-week-old female SCID mice under general anaesthesia (figure 1*a*). After eight weeks, the constructs were explanted. Before implantation (T0) and after explantation (T1), the constructs underwent mCT acquisition—in this way, we are able to show developments over time by comparing the same construct at two time points. To guarantee sterility and proper cell activity, the constructs were stored in sterile Eppendorf vials (Eppendorf AG) filled with embryonic stroma cell medium (DMEM high glucose with the addition of 12.5% FCS, 2 mM l-glutamine, 1% non-essential amino acids, 50 μM *β*-mercaptoethanol (Life Technologies, Carlsbad, CA, USA), 100 units ml^−1^ penicillin, 100 μg ml^−1^ streptomycin, 4 ng ml^−1^ basic fibroblast growth factor (Active Bioscience, Hamburg, Germany) and 2.5 μg ml^−1^ amphotericin B (Merck, Darmstadt, Germany) in T0 during acquisition. In T1, constructs were scanned in the same type of Eppendorf vials filled with PBS (Life Technologies).

### 2.4 Animal treatment

An important requirement for the housing of SCID mice is a specific pathogen-free environment which was ensured by the use of positive pressure isolators with stable conditions of 21^°^C and 75% humidity. The mice lived in groups of three animals in 320 cm^2^ macrolon cages (Tecniplast, Hohenpeissenberg, Germany). Bedding, food, water and environmental enrichment was sterilized prior to use within the cages. Health checks and control of temperature and humidity were performed once per day, the cages were changed once per week. After surgery, health checks were provided one, two, four, six, 12 and 24 h after implantation of the constructs. Mice were sacrificed by cervical dislocation.

### 2.5 Micro-computed-tomography acquisition parameters

mCT analysis was performed using a SkyScan 1076 Hasitom (Bruker, Billerica, MA, USA) at a tube current of 200 μA, an integration time of 420 ms per projection and a tube voltage of 50 kV_p_. Pixel size was 17.7 μm—referring to a voxel edge length of 17.7 μm. The voxels have to be isotropic to ensure the correctness of subsequent post-processing. A 0.5 mm aluminium filter was used for absorbing the low-energy part of the generated X-ray spectrum. Each projection was acquired seven times (frame averaging). We followed the advice for optimum acquisition as described by Bouxsein *et al.* [6]. To avoid artefacts, constructs were surrounded by liquid during acquisition as described above.

Three-dimensional reconstruction was done with NRecon (Skyscan, Kontich, Belgium), the mCT vendor’s recommended program, based on the acquired projections, which were saved in single tagged image file format (TIFF) files by the scanner. Reconstruction was performed using a standardized protocol: beam hardening correction=10, and ring artefacts reduction=6. Misalignment compensation was evaluated for all of the constructs individually and has been performed when necessary.

The reconstructed datasets—also TIFF files—were then loaded into the proposed program, the Heidelberg-mCT-Analyzer.

### 2.6 Histology

After explantation and mCT acquisition for T1, the constructs were histologically analysed by performing haematoxylin, eosin and safranin (HES; Carl Roth, Karlsruhe, Germany) staining according to standard protocols. For each construct, six slices out of three different levels were stained. The thickness of each slice was 5 μm, while the distance between the respective levels was 50 μm. Histological analysis was performed to prove qualitatively that there is bone formation within the construct (figure 1*c*).

### 2.7 The Heidelberg-mCT-Analyzer

In this study, we present a framework for fast and user-independent analysis of bone-like structures imaged with high-resolution mCT. The proposed algorithm, however, is not only limited to the analysis of those structures at one point in time, but also allows for the comparison of two time points for the same construct (e.g. before implantation and after explantation following a certain time *in vivo*).

In a first step, the construct’s three-dimensional reconstruction of the first point in time (T0) was segmented using fuzzy clustering with fully automatic level-setting [13]. The threshold obtained by this classification was then applied on the construct’s reconstruction of the first time point and the later time (T1) point. In a second step, the pores within the construct were extracted by means of the simplified bubble analysis, which is also used for biopolymer network pores [14]. In a third step, the segmented construct and pores from both points in time were analysed and quantified.

Post-processing was done in MATLAB R2013A (MathWorks, Natick, MA, USA) using the Curve Fitting Toolbox, the Fuzzy Logic Toolbox, the Image Processing Toolbox and the Statistics Toolbox. In addition to the original MATLAB functions, several custom algorithms were implemented. Those custom algorithms include functions for the scaffold segmentation (implementation of the fuzzy logic, evaluation of the clustering, artefact correction), the statistical analysis of the constructs (calculating the scaffold’s isosurface, calculating the scaffold’s convex hull, finding and analysing the pores within the scaffold) and the visualization of the results. The complete source code is available in the electronic supplementary material (Source Code).

### 2.8 Segmentation of the construct

**Figure 2.**
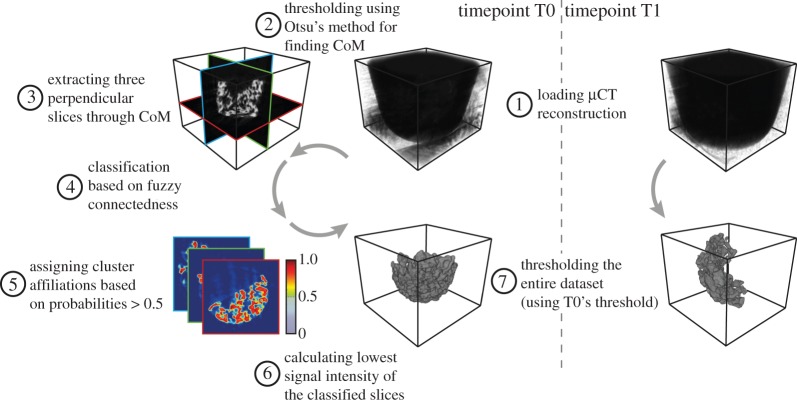
Schematic of the construct segmentation algorithm. After loading the three-dimensional *μ*CT reconstruction of time-step T0 (1), its CoM was calculated (2). The CoM represents the reference point for the extraction of three orthogonal slices used for the construct classification (3). For each voxel of these slices, cluster-affiliation probabilities were calculated based on the voxels’ connectedness in terms of signal intensity (4). By thresholding the cluster-affiliation probabilities, the classification was transformed from a continuous to a binary classification (5). The three orthogonal slices were then masked with the binary classification and the lowest signal intensity was obtained (6). This signal intensity was then used to threshold the complete three-dimensional *μ*CT reconstruction (7). To be able to compare T0 and T1 of the same construct, T1 was segmented using the same threshold as T0.

The reconstruction of T0 was loaded slice per slice and saved in a three-dimensional volume (figure 2(1)). The construct’s segmentation was done in two steps—first, automatic selection of three representative slices of the volume, and second, obtaining a threshold that represents the characteristic intensity and the ‘hanging togetherness’ based on the local signal intensity homogeneity [15]. The centre of mass (CoM) of the construct was determined as a reference point for the representative slices. In order to calculate the CoM, prior to the slice extraction, a global threshold was computed based on Otsu’s method [16] and then applied on the whole reconstruction (figure 2(2)). These three representative, and perpendicular slices were extracted in transversal, sagittal and coronal orientation intersecting at the CoM to cover the whole extent of the dense parts of the construct (figure 2(3)). To benefit from the ‘adaptive’ thresholding performed by fuzzy clustering [17] and to overcome its long computational time when applied on a whole three-dimensional dataset [18], we decided to use only those representative slices for the fuzzy clustering. For each voxel of the three slices, a probability was calculated to be part of either the cluster *construct* or the cluster *non-construct* (figure 2(4)). The fuzzy clustering, however, was performed twice for each voxel to minimize emergent phenomena. For further calculations, the higher cluster probability was taken into account. A binary mask was created for each of the slices based on a voxel’s affiliation to the cluster *construct*—all voxels showing a probability of the cluster *construct* greater than 0.5 were characterized as *true*, all the others as *false* (figure 2(5)). After multiplying the binary masks with the corresponding slices, the lowest signal intensity of each slice was obtained (figure 2(6)). Averaging these three intensities resulted in the global threshold used for the segmentation of the whole construct within the three-dimensional mCT reconstruction. Since our approach was to segment the construct not only at one but also at a second point in time, the global threshold calculated for T0 was also applied on T1 (figure 2(7)). Prior to pore extraction and quantitative analysis of the construct and the pores, the segmented constructs were corrected for artefacts. To reduce the amount of isles with only a few voxels, all subvolumes contributing less than 0.1% to the total volume were removed. The segmented as well as the original volumes and the corresponding threshold were then saved in a MATLAB variable for subsequent post-processing. T0 and T1 were processed equally during subsequent analyses.

### 2.9 Extraction of the pores

**Figure 3.**
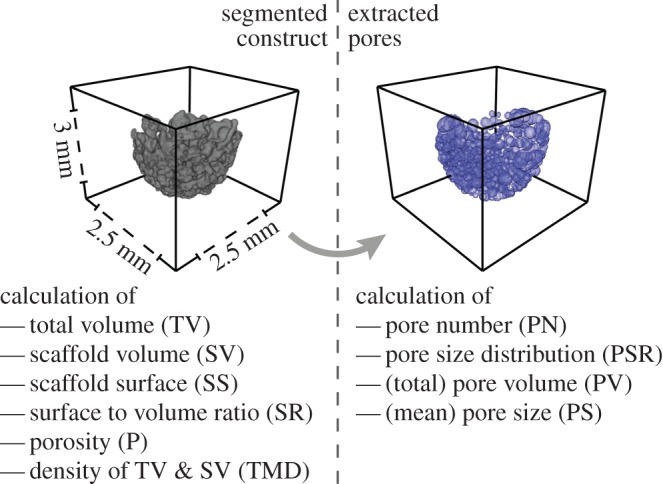
Calculated parameters from the segmented construct and the extracted pores and final size of the scaffolds. Pores were extracted based on the EDM of the fluid space of the construct and the EDM’s local maxima. The obtained parameter set contains absolute values (TV, SV, SS, P, PV), mean values±s.d. (PS, TMD) and distributions (TMD, PSR). The dimension of the constructs is shown representatively.

After successful segmentation, the construct’s pores were extracted. The proposed algorithm is based on approaches used for the analysis of biopolymer networks [14,19]. Since the pores in the tissue-engineered construct were shaped irregularly, the pore size was determined by calculating the radius of the largest sphere fully contained in the non-construct phase. First, after preprocessing the three-dimensional mCT reconstruction slice per slice, by masking the dataset into construct voxels with value 1, non-construct voxels within the construct’s convex hull with value 0, and all non-construct voxels outside this convex hull with ‘not-a-number’ (NaN), the Euclidean distance map (EDM) was calculated for the non-construct voxels within the hull. The EDM shows each non-construct voxel’s Euclidean distance to the nearest construct voxel. By identifying local maxima, it was possible to draw conclusions on the location as well as on the diameter of each single pore (figure 3, right). Although this method was adapted from a two-dimensional approach, it holds true for three-dimensional analyses [14].

### 2.10 Construct and pore analysis

Knowledge of the construct’s properties and its pore size distribution is crucial for understanding the construct’s behaviour *in vivo*, because these parameters influence the scaffold’s mechanical properties, the diffusive transport within the construct and cell migration through such bone-like structures [4,5,20].

The following parameters were obtained from the segmented construct (figure 3, left):

— *Scaffold volume* (SV). This parameter represents the amount of voxels that were automatically segmented from the entire mCT reconstruction as a construct.
— *Scaffold surface* (SS). First, the faces and vertices of the isosurface of the segmentation’s results were calculated. Then the faces and vertices were vectorized and transformed into patches and their corresponding polygonal description. Adding up all the polygons’ areas resulted in the surface of the segmented construct.
— *Scaffold surface to volume ratio* (SR). This parameter measures the ratio of scaffold surface to scaffold volume (SR=SS/SV). An increased surface area to volume ratio of the construct results in an increased exposure to its environment.
— *Porosity* (P). This parameter represents the ratio of a material’s total volume to the volume of empty spaces in the material itself (*P*=1−SV/TV).
— *Total volume* (TV). For obtaining the total volume of the construct, the convex hull of the construct was calculated based on a Delaunay-triangulation of the voxels classified as construct. All voxels within the convex hull represent the total volume.
— *Tissue mineral density* (TMD). The density distribution as well as the average density with its standard deviation were used to calculate the total volume (TV) and the scaffold volume (SV). Therefore, all voxels within the convex hull (in case of the density of TV), respectively, all voxels of the construct segmentation (in the case of the density of SV), were analysed.

Prior to the analysis of the pores, the smallest and the largest 5% of the pores were removed from the distribution given by the pore extraction algorithm described above. This data selection was performed to remove outliers and increase the robustness of the analysis.

The following parameters were then obtained from the (corrected) pore size distribution (PSR) (figure 3, right):

— *Pore number* (PN). This parameter represents the total amount of pores.
— *Pore size* (PS). PS represents the average pore diameter of the distribution and its standard deviation.
— *Pore volume* (PV). Instead of using the delta between TV and SV as pore volume, we decided to approximate each pore as a sphere and calculate the sphere’s volume based on the pores’ diameter. PV represents the sum of all spherical volumes.

All calculated parameters were saved as MATLAB variables along the (segmented) constructs for subsequent analysis.

### 2.11 Advanced statistics based on previously calculated parameters

The workflow described so far was only focusing on one construct (although two points in time, T0 and T1, were evaluated)— additional calculations, however, were performed for a large set of constructs to be able to compare T0 and T1 on a statistically significant basis.

The following features were obtained and compared within the mCT-Analyzer:

— *Histogram of the pore size distribution*. For T0 and T1, the pore size distributions of all constructs were merged. For each time step, a pore size histogram was created. As a guide to the eye, the delta between T0’s and T1’s histogram was calculated and displayed (see the electronic supplementary material, figure S1 (left)).
— *Slope of the empirical cumulative distribution function of the pore size distribution*. Based on the histograms above, the cumulative distributions were calculated (see the electronic supplementary material, figure S1 (right)). Afterwards, a linear function was fitted to the first-half of the bins of the logarithmized distributions. We decided to analyse only this first subset exemplarily within this publication. In general, the steeper the slope of the linear fit, the bigger the contribution of the small pores to the distribution. The algorithm, however, is not limited to this first subset. The pore size range used for the linear fit is customizable to suit the scientific question.
— *Histogram of the grey-scale distribution* (in Hounsfield units, HU). The HU distributions for T0 and T1 of all constructs were combined. Only voxels that had been segmented by the fuzzy clustering algorithm were analysed. For each time step, a HU histogram was created. Similar to the pore size histograms, the delta between T0 and T1 was calculated and displayed (see the electronic supplementary material, figure S2).

Not all parameters obtained from the single constructs were used for advanced calculations within the mCT-Analyzer. However, they are saved in human-readable files, and therefore can be processed with, for example, SPSS or R, or any other equivalent software. Since the algorithm was written in a modular way, further custom analysis can easily be added to the mCT-Analyzer.

### 2.12 User interaction

User interaction is only needed during three subroutines of the mCT-Analyzer:

— during the selection of the datasets;
— after the segmentation for evaluation purposes, to decide if the construct should be further processed to calculate the parameters described above; and
— after the analyses of the merged datasets, to choose where to save the output.

In terms of user-friendliness, reducing the user’s influence on the results was not the only requirement, but also minimizing the total time a user has to spend interacting with the software while analysing multiple constructs. Therefore, in a first step, the user selects all desired constructs, the constructs are segmented automatically (see ‘Segmentation of the construct’) and are then presented for evaluation. In a second step, the mCT-Analyzer executes all routines requiring extensive computational power and time (see ‘Extraction of the pores’ and ‘Construct and pore analysis’) for all constructs and time points automatically without user interaction. In a third and final step, the mCT-Analyzer summarizes all calculations (see ‘Advanced statistics based on previously calculated parameters’), presents the results obtained from the analyses of all constructs and provides the possibility to save them as text-files, image-files and MATLAB variables.

### 2.13 Algorithm evaluation

To ensure that the results obtained with our proposed algorithm are reliable, we compared a subset of those results with the results acquired with Amira (FEI Visualization Sciences Group, Bordeaux, France). The pore size is one of the core parameters of our study, therefore we review only this parameter in detail (other parameters can be found in the electronic supplementary material). Since Amira’s Quantification module uses a different algorithm to determine the size of single pores, we compared the total volume of all pores. The percentage of deviation was calculated by the following formula:

$$\text{deviation}=\frac{0.5-\text{PV}(\text{HD}-\text{mCT})}{\text{PV}(\text{HD}-\text{mCT})+\text{PV}(\text{Amira})},$$

with PV(HD−mCT) representing the total pore volume calculated with our algorithm and PV(Amira) representing the total pore volume calculated with Amira. For the ideal case when PV(HD−mCT) and PV(Amira) are equal, the deviation is zero. For PV comparison, we used 10 randomly chosen constructs (from T0 and T1).

### 2.14 Statistics

Statistical analysis for comparing the two points of time (T0 and T1) was done using the paired two-sample *t*-test for normally distributed samples and the Wilcoxon signed-rank test for not normally distributed samples. Normality of the samples was tested with the Shapiro–Wilk test.

Results were deemed statistically significant if *p*<0.05. Statistical analyses were done with SPSS Software (IBM Corporation, Armonk, NY, USA). Boxplots were created with Sigmaplot software (Systat Software Inc., San Jose, CA, USA).

## 3. Results

In order to show potential differences in construct properties related to bone formation over time, we analysed and compared construct properties of T0 and T1 using our novel mCT analysis algorithm. In addition to basic scaffold parameters, we also obtain complex parameters, such as entire distributions, for example, of the pore diameters. This is done to enhance the scaffold characterization, because several *in vivo* properties cannot only be described by a mean value and its standard deviation.

### 3.1 Evaluation of TV, SV, SS and PV

**Figure 4.**
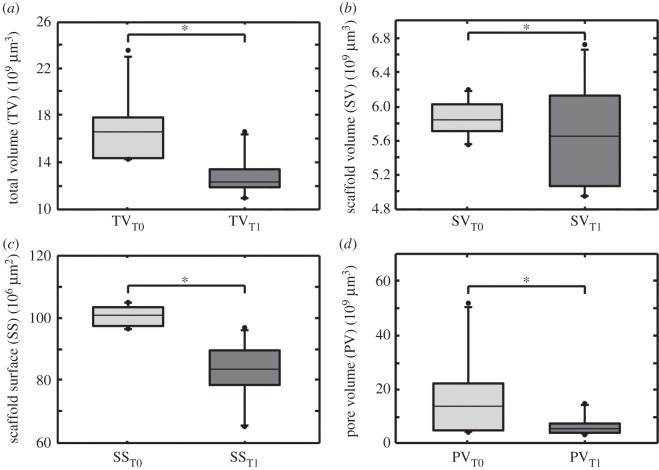
Boxplots illustrating change of total volume (TV), scaffold volume (SV), scaffold surface (SS) and pore volume (PV) over time. Boxplots are designed as median±interquartile range (IQR); range from highest and lowest value within 75th percentile±1.5 IQR; points are illustrating outliers. (*a*) TV at T0 compared to TV at T1. Differences were significant (*p*<0.0001); (*b*) SV at T0 compared to PN at T1. Differences were not significant (*p*=0.219); (*c*) SS at T0 compared to SS at T1. Differences were significant (*p*<0.0001); (*d*) PV at T0 compared to PV in T1. Differences were significant (*p*=0.0439).

**Figure 5.**
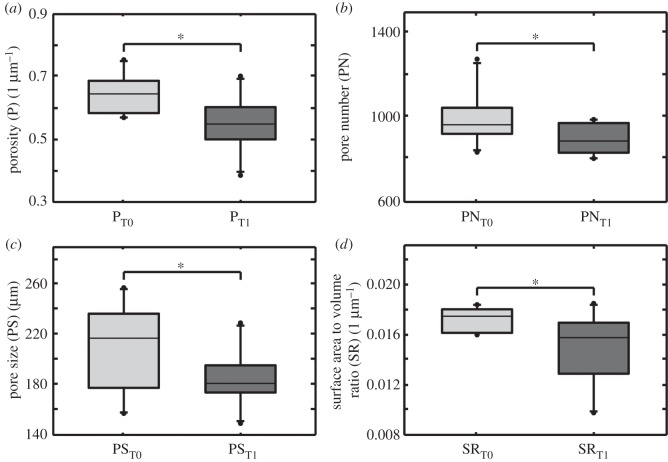
Boxplots illustrating change of porosity (P), pore number (PN), pore size (PS) and surface to volume ratio (SR) over time. Boxplots show median± interquartile range (IQR); range is from highest and lowest value within 75th per cent value±1.5 IQR; points illustrate the maximum and minimum outliers. (*a*) *P* at T0 compared to *P* at T1. Differences were significant ( *p*=0.001); (*b*) PN at T0 compared to PN at T1. Differences were significant ( *p*=0.013); (*c*) PS at T0 compared to PS at T1. Differences were significant ( *p*=0.032); (*d*) SR at T0 compared to SR in T1. Differences were significant ( *p*= 0.007).

TV decreased significantly from T0 to T1 from an average of 1.69×10^10^μm^3^ to 1.28×10^10^μm^3^, i.e. a decrease of 24.2% (figure 4*a*). SV decreased from a T0 level of 5.86×10^9^μm^3^ to a T1 level of 5.65×10^9^μm^3^, which means a decline of 3.6%. Differences were not significant (figure 5*b*). We calculated the coefficient of variation (CoV) for SV in T0 to proof the identical shape and size of the constructs. We choose SV as it represents the exact dimensions of the construct. CoV for SV in T0 is 3.41%. The surface of constructs (SS) decreased from an initial area of 1.01×10^8^μm^2^ to 0.83×10^8^μm^2^ in T1. Differences were significant (figure 4*c*). PV decreased significantly from a T0 level of 1.70×10^10^μm^3^ to 0.635×10^10^μm^3^ in T1, i.e. a decline of 61.7% (figure 4*d*). For significance levels, see table 2.

**Table 2.** Significance levels for the parameters’ development over time. The normality of the samples was tested with the Shapiro–Wilk test. Afterwards, according to the normality, the appropriate statistical test was performed to quantify significance level *p* (results are significant for *p*<0.05).

parameter	distribution	statistical test	significance level *p*
TV	not normal	Wilcoxon	0.005
SV	normal	*t*-test	0.219
SS	not normal	Wilcoxon	0.005
SR	not normal	Wilcoxon	0.007
P	normal	*t*-test	0.001
PN	not normal	Wilcoxon	0.013
PV	not normal	Wilcoxon	0.009
PS	normal	*t*-test	0.032

### 3.2 Evaluation of P, PN, PS, PSR and SR

P decreased significantly from 0.645 μm^−1^ in T0 to 0.55 μm^−1^ in T1 (figure 5*a*). PN decreased significantly from an average number of 988 in T0 to 894 in T1. That means a median decrease of 94.3 pores (9.55%) over time (figure 5*b*). From a T0 level of 207 μm, PS declined significantly to 185 μm in T1, which means a decrease of 10.8% (figure 5*c*). SR also decreased significantly from 0.017 μm^−1^ in T0 to 0.015 μm^−1^ in T1, which means a decrease of 12.2% (figure 5*d*). For significance levels, see table 2.

Furthermore, we analysed PSR in steps of 0–100 μm, 100–200 μm, 200–300 μm, 300–400 μm, 400–500 μm and 500–600 μm pore diameter according to the suggestions from Karageorgiou & Kaplan [5].

We observed significant declines in the range of 200–300 μm and 500–600 μm as well as non-significant declines within the range of 0–100 μm and 400–500 μm. In the area of 300–400 μm, we found a non-significant increase of pores from T0 to T1 (table 3).

**Table 3.** Diameter of pores within the set range. Pores with a diameter of more than 600 μm are not mentioned within the table. PN T0 and PN T1 show the average number of pores per construct within the set range for T0 and T1, respectively. Δ(1−T0/T1) is the difference in percentage of pore numbers at T0 and T1. The *p*-value shows significances after paired *t*-testing as described in section ‘Statistics’, *p*<0.05 is set as significant.

diameter (μm)	[0, 100)	[100, 200)	[200, 300)	[300, 400)	[400, 500)	[500, 600)
PN T0	204	398	228	78	31	18
PN T1	199	383	188	86	27	5
Δ(1−T0/T1) (%)	−2.36	−3.84	−21.20	9.31	−15.30	−270.83
*p*-value	0.704	0.219	<0.001	0.116	0.329	<0.001

For analysis and discussion of TMD, see the electronic supplementary material.

In histology, bone formation has been observed in every construct (figure 1*c*).

### 3.3 Evaluation of the algorithm

Comparing PV calculated with our algorithm and calculated with Amira demonstrated that our algorithm overestimated the total pore volume 3.63±4.91% (*n*=10). The comparison of P and SR showed that there were no differences between Amira and our algorithm, because the calculations are based on the same segmented constructs. Whereas PV, P and SR showed no differences, PN differed.

Since our algorithm requires user input only in terms of sample selection and not in terms of post-processing decisions (e.g. threshold), the inter-user reliability is very high. Multiple users obtained the same results for the same constructs.

We furthermore assessed the computational time for the calculations. See the electronic supplementary material for the results.

## 4. Discussion

In this study, we built 10 constructs from hMSCs of five different patients and added 1 μg ml^−1^ BMP-7 for stimulation of bone formation [21]. We performed mCT acquisition before implantation (T0) and after explantation (T1) and compared the results of both time points.

According to our established tissue-engineering model, we were able to show ectopic bone formation of human MSCs in SCID mice [22]. Histochemistry was the gold standard for the detection of de novo bone formation [8]. During the last years, however, mCT has become important for evaluation of bone microstructure and bone formation [7,23]. Although mCT is well established for the evaluation of bone structure, for the analysis of scaffold properties it is not commonly used as yet.

We present a new, easy-to-use analysis tool, focused on evaluation of scaffold properties in bone tissue engineering. The Heidelberg-mCT-Analyzer allows a fast, accurate, reliable and user-independent analysis of scaffold microstructure. Our open-source software script allows modifications for individual adaption to multiple study designs and settings focusing on scaffold analysis.

The following parameters correlating with bone formation and interaction within implant and host can be extracted using mCT [6]:

— Change of implant volume over time (TV and SV).
— Change of implant surface over time (SS).
— Change of surface area to volume ratio over time (SR).
— Change of pore size, pore number, pore volume and porosity over time (P, PN, PV, PS).
— Change of implant density over time (TMD).

### 4.1 TV and SV

We used bTCP as the matrix of our implanted scaffold. bTCP is known to have a high biodegradability. Within 16 weeks, about 40% of bTCP was resorbed in a trial with minipigs [24]. Therefore, we also expect seeing a reduction of the bTCP volume during our implantation period of eight weeks. We used the parameters TV and SV to illustrate the volume change over time. SV refers to the volume of the segmented scaffold without the pores within this construct. SV, therefore, consists only of voxels with a signal intensity above the automatically obtained threshold. TV is the volume of all voxels within the scaffold’s convex hull. TV, therefore, consists of SV and the voxels of the pores within this scaffold.

TV decreased significantly from T0 to T1. SV showed a small decline from T0 to T1, although changes were not significant.

We believe that the time of implantation was too short to detect a significant resorption of bTCP, represented mainly by SV, which is also indicated in the literature [24]. Furthermore, we implanted the bTCP constructs ectopically in subcutaneous pouches. Compared to implantation in bone, vascularization and cell migration are lower. However, the scaffolds were seeded with MSCs. They stimulate the invasion of osteoclasts, which means that the resorption is mainly supported by cells that have to invade the construct [25].

The significant decrease of TV is a result of the design of the parameter itself: the total volume of interest (VOI) includes ‘soft’ structures such as glue, cells, cell medium and liquid. Those materials are not very stable in their integrity and are highly bio-resorbable compared to bTCP [26,27]. Even within a short time of implantation, the surrounding of the bTCP is resorbed properly.

These data imply that there are active processes of resorption during the implantation period. Resorption is an early sign of bone formation, caverns are replaced by newly formed bone tissue [28]. Also, compared to the histological data presented, one can clearly see bone formation within excavations and surface of the bTCP (figure 1*b*).

### 4.2 SS

The parameter SS shows alterations at the surface of the scaffold. On the one hand, change in scaffold surface could be induced by resorption processes; on the other hand, de novo formation of bone is able to both expand and shrink the scaffold surface. SS was also calculated after segmentation. SS decreased significantly from T0 to T1.

### 4.3 P, PN and SR

P, PN and SR are important scaffold characteristics and play an important role for bone ingrowth [5,20]. mCT is a very reliable and precise method of quantifying P, PN and SR of a selected scaffold structure. Besides counting PN, Heidelberg-mCT-Analyzer also detects PS and PV. Pore configuration and especially an adequate pore diameter are important factors for successful bone formation and are controversially discussed in scaffold design, development and research [5,20,29]. Literature indicates no formation of calcified tissue is possible at a diameter below 100 μm, especially because of the lacking vascularization of small pores [5,30]. Nonetheless, the role of pore diameter has not yet been clarified and remains controversial [5,29]. Therefore, the Heidelberg-mCT-Analyzer is able to show the number of pores of a selected size (PSR). The bins are automatically set using a width of 10 μm but can also be adjusted according to specific needs. This helps to analyse the optimum pore size for the stimulation of bone ingrowth into the scaffold when evaluating the changes of pores over time. Our results demonstrate that PN significantly decreases significantly over time. This is also represented by the decrease of SS—porosity is one of the substantial factors for surface expansion. If pores disappear because of new bone filling, SS decreases as well.

### 4.4 PV, PS and PSR

The significant decrease of PV from T0 to T1 of more than 61% emphasizes that pores get filled with new bone. De novo filling of pores with bone could also be observed in histology (figure 1*c*). The same happens to PS, which declines significantly by more than 47% from T0 to T1. SR also decreased significantly from T0 to T1—so did P, which means that the construct became more compact and dense over time because of tissue formation and ingrowth in formerly free space.

**Figure 6.**
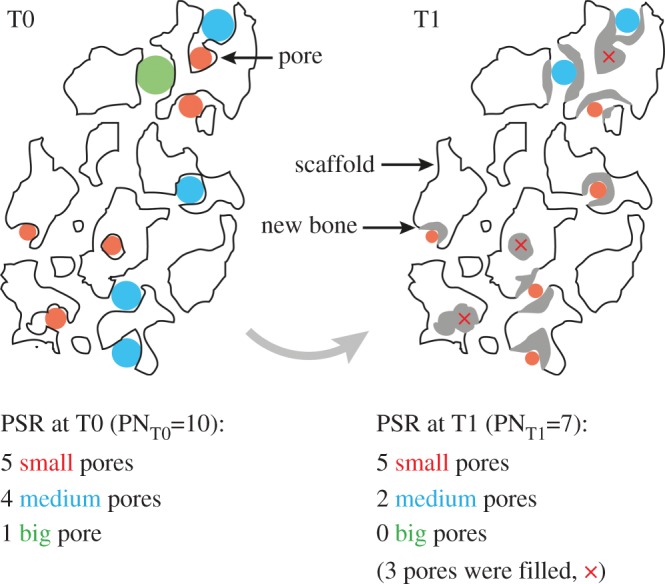
Graphical representation of the idealized pore size range (PSR) of one single slice of a scaffold for time points T0 and T1. Pores and pore sizes are colour-coded. The scaffold’s outline is shown in black. Newly formed bone material is represented in grey. Since this is an idealized representation, not all pores and newly formed bone fillings are displayed. Comparing T0 and T1 showed that the total pore number (PN) decreases due to bone formation. In this example, only the number of big- and medium-sized pores were affected by the newly formed bone material—the amount of small pores did not change over time. Analysing each pore individually, however, showed that nearly all pores were affected by the bone formation.

A decline of the number of pores with a certain diameter could be interpreted as bone ingrowth and bone formation within that subset of pores [4,5,20,29]. PSR of T0 and T1 (table 3) showed different behaviour for different pore size ranges: a decline of the number of pores (subsets 200–300 μm and 500–600 μm), as well as no significant changes (subsets 0–100 μm, 100–200 μm and 400–500 μm), as well as increasing pore numbers (subset 300–400 μm). These contradictory findings can be explained by detailed observation of the pore behaviour over time (figure 6): bone growth within the smallest subset of pores is able to fill the pore completely, so the pore ‘disappears’ in T1. Within the subset representing the number of pores with the next larger diameter, bone formation is not able to close the pore but does reduce the size of the pore—as a consequence, the pore is counted within the smaller subset of pores in T1. This could cause both an artificial equilibrium state in one subset of pores or even an increase of the count of pores.

### 4.5 Assessment

The behaviour of the parameters over time have to be assessed in common: the changes in TV and SV can be compared to those in SS. The surface of SS decreases within the set threshold because of resorption processes which start at the surface of the construct and proceed to the more central structures and get filled with newly formed bone tissue supplied by osteoblasts in the rear guard [24,28]. Furthermore, the constructs are exposed to mechanical stress caused by the movement of the animals after surgery. Even though our bTCP scaffolds are solid, mechanical stress compresses the bonded parts of the construct and will artificially reduce the scaffold’s surface. The changes in TV, SV and SS indicate a decrease in high-dense structure surface, which means that the bTCP gets reabsorbed and the developing lacunae are filled with new bone tissue (figure 1*b*) [28].

The pore-related parameters (P, PN, SR, PS and PV) show the same development: the total number of pores (PN) decreases, as does the PV, SR and P itself. Pores are filled by newly developed bone. PS can detect the most relevant group of pores for bone ingrowth—our results are in agreement with already published data [5].

In summary, changes in the structure of the implanted constructs over time by mCT assessment could be detected. The standard parameters mentioned (table 1) have been described as being crucial for bone development and change over time if bone formation takes place, and are well established [6,8,20]. Furthermore, due to analysis of further parameters such as the pore size range, the changes within the scaffold over time could be described in even more detail (table 1). Through histological analysis of the constructs, which is currently the gold standard in tissue engineering of bone, we could clearly demonstrate that there is bone formation within the scaffolds. The changes detected by mCT therefore represent the changes caused by bone formation.

Comparing our approach with already established methods (in this case, Amira) not only showed that our algorithm was capable of analysing a bigger set of parameters but also confirmed that our algorithm creates comparable results. Comparing widely used parameters such as pore volume (PV), porosity (P), scaffold surface to volume ration (SR) and pore number (PN) showed that our algorithm obtained the same PV, P and SR, but identified a different PN. This was due to different algorithms used for the estimation of pore sizes. We used an analysis which fits spheres in the automatically segmented non-scaffold subvolumes. Amira separated the pores by means of computing watershed lines on the manually thresholded grey-level image. Therefore, PN calculated by Amira was lower than PN obtained by our algorithm. Although the pores of the scaffolds are usually rarely spherical, using the covering radius transform is the most reproducible way to quantify pore sizes due to its robustness and standardization. Furthermore, it enables us to assess the pore size distribution, and therefore allows us to analyse not only the volume of all pores but also the volume of each single pore [14,15].

Furthermore, comparing the Heidelberg-mCT-Analyzer evaluation of P in T0 to the manufacturers’ datasheet showed that our results were plausible. A possible explanation for the higher grade of porosity measured in our setting is provided by the scaffold preparation protocol: by sticking the bTCP granules together, new pores occurred which are not located inside the bTCP but among the granules (figure 1*b*). The manufacturers’ data provide only the pores within the bTCP granules.

In this study, we were able to demonstrate that the Heidelberg-mCT-Analyzer is capable of analysing the major properties for osseointegration, osteoconduction and osteostimulation of scaffolds. Our analysing software is, therefore, a promising tool for standardized evaluation of scaffold properties in tissue engineering. Particularly because of the possibility of three-dimensional viewing, the size, the change of volume and structure of pores could be observed in detail [7,8,23].

Although the changes of parameters P, SR, PV, PN and PS are established as being correlates of bone formation over time, histology stays the gold standard for the evaluation of bone formation because of the better differentiation of bone and other tissues. The idea of fitting mCT pictures onto histology slices for a precise comparison of structure and bone formation should be evaluated in further studies. This would combine the advantages of mCT (precise calculation of changes, volume rendering, etc.) and histology (exact differentiation of tissues, high image resolution) in order to evaluate bone structure and bone formation more accurately. However, standardized protocols of acquisition and reconstruction are necessary to minimize bias in the evaluation by the Heidelberg-mCT-Analyzer that could probably affect the results. Therefore, we propose to follow the present recommendations [6].

As a perspective, with further validation and application of our protocol, the number of histological examinations could be reduced, and at least partially replaced by mCT analysis. Especially in protocols observing scaffold behaviour and properties *in vivo* over time, an intensified use of mCT in combination with the Heidelberg-mCT-Analyzer as a non-destructive approach can deliver objective three-dimensional data and also help to reduce the number of experimental animals needed.

## 5. Conclusion

In this study, we presented the Heidelberg-mCT-Analyzer—a fast, accurate, reliable and user-independent tool for the fully automatic evaluation of scaffold structure in bone tissue engineering. In a proof of concept experiment, we were able to analyse ectopic bone formation of hMSC-coated bTCP stimulated by BMP-7 in SCID mice over time. Furthermore, we could confirm the algorithm’s results by comparing them with already established tools. In addition to basic parameters describing the tissue-engineered scaffolds, we obtained more complex parameters, such as the entire distribution of pore diameters and tissue mineral densities. Because of the algorithm’s modular design, it allows for individual modifications enhancing multiple tissue-engineering protocols and their quantitative analyses.

## Supplementary Material

20151012 Supplementary Material.docx

## Data accessibility

The data used in this publication are stored on Dryad: http://dx.doi.org/10.5061/dryad.6nv82.
