# Supplementary material for: Heidelberg-mCT-Analyzer: a novel method for standardized microcomputed-tomography-guided evaluation of scaffold properties in bone and tissue research
Source: R Soc Open Sci. 2015 Nov 11;2(11):150496. doi: 10.1098/rsos.150496 (PMC4680623; doi:10.1098/rsos.150496)
Supplement: 20151012 Supplementary Material.docx [file rsos150496supp1.docx]

**Supplementary Material (SM) of**

Heidelberg-mCT-Analyzer: a novel method for standardized micro-computed-tomography-(mCT)-guided evaluation of scaffold properties in bone and tissue research.

**METHODS**

*hMSC characterization, isolation and cultivation:* Bone marrow aspirate was taken from the anterior iliac crest of every patient. The isolation and definition of hMSC was performed as described previously [1, 2]. Cultivation was performed in gelatinized T75 cell culture flasks under the use of embryonic stroma cell (ES)-medium (DMEM high glucose with the addition of 12.5% FCS, 2 mM L-Glutamine, 1% non-essential Amino Acids (NEAA), 50 µM β-Mercaptoethanol (Life Technologies, Carlsbad, CA), 100 units/ml Penicillin, 100 µg/ml Streptomycin, 4 ng/ml basic Fibroblast Growth Factor (bFGF) (Active Bioscience, Hamburg, Germany), and 2.5 µg/ml Amphotericin B (Merck, Darmstadt, Germany) at 37 °C and 5% under standardized sterile conditions. After confluence of more than 80%, cells isolated from primary material were frozen as passage-zero-cells in liquid nitrogen.

After thawing according to common protocols, cells were cultivated as described up until passage two, and then used for scaffold coating after 80% of confluence.

*Histomorphometric analysis:* The quantitative analysis of the histological slides was performed by the use of Adobe Photoshop CC 14.0 (Adobe Systems Incorporated, USA). The HE-stained slides were digitized, then the total area of the construct was labeled in black while the area of the newly formed bone was labeled in gray. With assistance of ImageJ 1.47v (Wayne Rasband National Institutes of Health, USA) it was now possible to calculate the percetage rate of the gray pixels related to the number of black pixels.

**RESULTS**
*Mice demographics*: the mice had an average weight of 20.7g (mean (SD)=0.84). No adversal events occurred.

*Histomorphometric analysis:* The average area of newly formed bone within the constructs was 4,95% (±4,66%).

*Computational times:* All calculations were done on a 64bit Windows 7 SP1 computer equipped with an Intel Core i7-3960X @ 3.3 GHz and 32 GB RAM. The mCT-Analyzer was programmed to adapt to the given RAM capacity – however, 16 GB as a minimum RAM size is recommended. Following computational times were needed for the samples we analyzed as a proof of concept.

- ‘Segmentation of the construct’:

~30 s for a construct with the size of 300 x 300 x 250 voxels

~5 s for adapting the threshold and corrections to the later time point.

- ‘Extraction of the pores’ and ‘Construct and pore analysis’:

~200 s (100 s for T0 + 100 s for T1) for a construct with the size of 300 x 300 x 250 voxels

- ‘Advanced statistics based on previously calculated parameters’:

~ 10 s per construct with the size of 300 x 300 x 250 voxels

Further analysis outcomes obtained by Heidelberg-mCT-Analyzer are shown in SM Fig. 1 and 2.


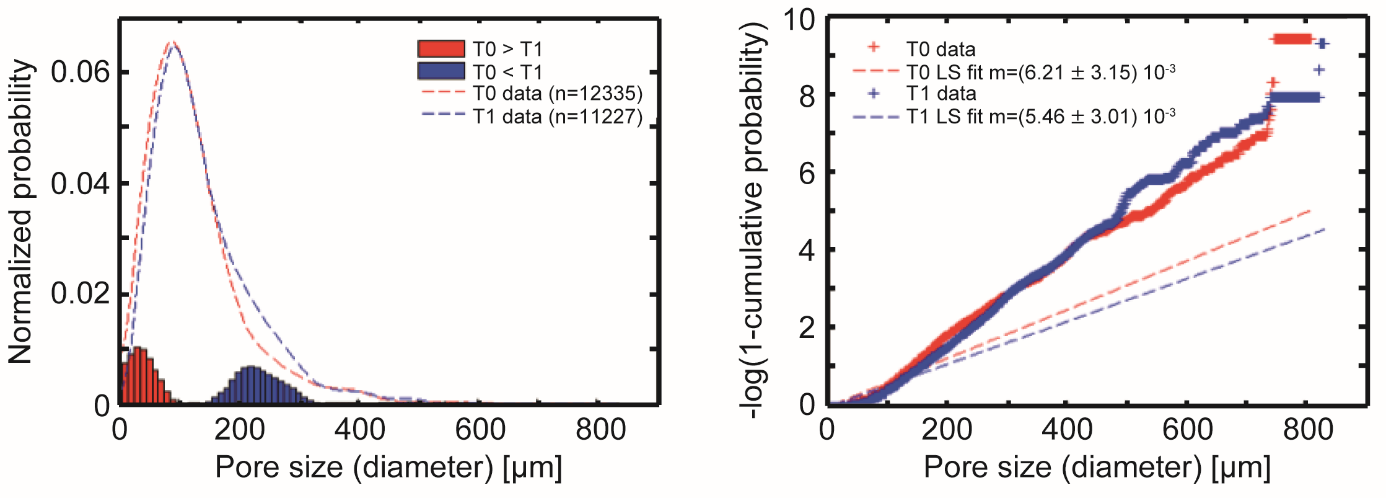


**SM Figure 1:** Analyses of the pore sizes of all constructs for T0 and T1.

LEFT: Histogram of all pore-diameters. The red curve represents T0, the blue curve T1. The bar plots show in corresponding color which time point’s contribution dominated for each bin of the pore size distribution. In this example, constructs of T0 show more pores < 100µm than the same constructs for T1. Pores > 100µm but < 300µm are found more often in the constructs measured at T1.

RIGHT: Logarithmized empirical cumulative distribution function (ECDF) of the pore-diameters. The red curve represents T0, the blue curve T1. The dashed lines represent a linear fit to the ECDFs for pore diameters < 500µm. The slope of the fit is a parameter representing the contribution of small pores to the whole pore size distribution. In this example, the small pores’ contribution to T0’s distribution is higher than to T1’s distribution, but there was no significant difference.


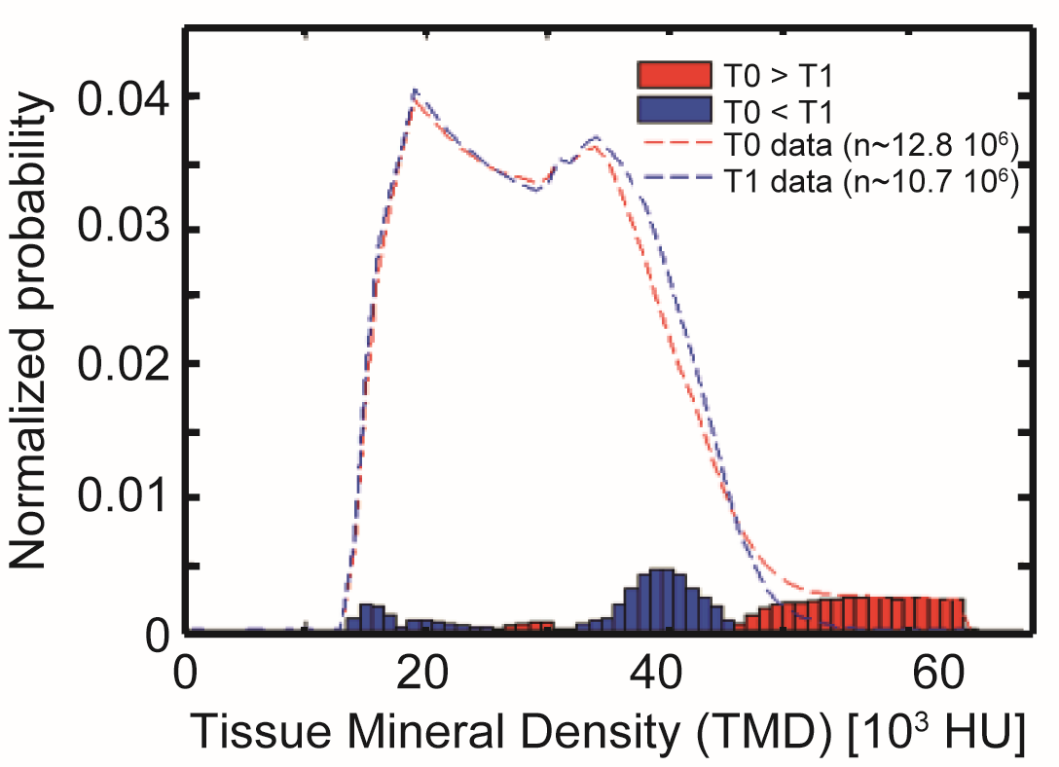


**SM Figure 2:** Histogram of the gray-scale distribution (in Hounsfield units, HU) of all segmented constructs for time point T0 and time point T1. The red curve represents T0 and the blue curve T1. As already described for Fig.1, the barplots show which bin of the histogram is dominated by which time point (red: T0 > T1, blue: T0 < T1).

**SOURCE CODE**

The source code (written in MATLAB) is available on request.

Please contact: [fabian.westhauser@med.uni-heidelberg.de](mailto:Heidelberg-mCT-Analyzer@med.uni-heidelberg.de) or [christian.weis@med.uni-heidelberg.de](mailto:christian.weis@med.uni-heidelberg.de)

For questions and remarks, please contact Christian Weis ([christian.weis@med.uni-heidelberg.de](mailto:christian.weis@med.uni-heidelberg.de)). The software comes as is, therefore, dedicated support cannot guaranteed.

**REFERENCES (SM)**

[1] Dickhut, A., Pelttari, K., Janicki, P., Wagner, W., Eckstein, V., Egermann, M. & Richter, W. 2009 Calcification or dedifferentiation: requirement to lock mesenchymal stem cells in a desired differentiation stage. *Journal of cellular physiology* **219**, 219-226. (doi:10.1002/jcp.21673).

[2] Dominici, M., Le Blanc, K., Mueller, I., Slaper-Cortenbach, I., Marini, F., Krause, D., Deans, R., Keating, A., Prockop, D. & Horwitz, E. 2006 Minimal criteria for defining multipotent mesenchymal stromal cells. The International Society for Cellular Therapy position statement. *Cytotherapy* **8**, 315-317. (doi:10.1080/14653240600855905).
